# Social Support and Manifest Anxiety as Predictors of Somatic and Cognitive Anxiety Symptoms in Children with Lower Leg and Ankle Fractures: A Cross-Sectional Study

**DOI:** 10.3390/healthcare13131569

**Published:** 2025-06-30

**Authors:** Goranka Radmilović, Marija Trconić, Martina Kolak Jurić, Marin Mamić, Ivan Vukoja, Dalibor Divković

**Affiliations:** 1 General County Hospital Požega, Osječka 107, 34000 Pozega, Croatia; goranka.radmilovic@pozeska-bolnica.hr (G.R.); ivan.vukoja@pozeska-bolnica.hr (I.V.); 2 Faculty of Medicine, Josip Juraj Strossmayer University of Osijek, J. Huttlera 4, 31000 Osijek, Croatia; divkovic.dalibor@kbo.hr; 3 Faculty of Medicine, University of Zagreb, 10000 Zagreb, Croatia; 4 University Hospital Centre Osijek, J. Huttlera 4, 31000 Osijek, Croatia; marija.trconic@kbco.hr (M.T.); martina.kolakjuric@kbco.hr (M.K.J.); 5 Faculty of Dental Medicine and Health Osijek, Josip Juraj Strossmayer University of Osijek, Crkvena 21, 31000 Osijek, Croatia

**Keywords:** anxiety, ankle fractures, children, lower leg fractures, social support

## Abstract

**Background:** Despite the high incidence of surgical treatment of lower leg fractures in children, there is little research focusing on the emotional consequences of such trauma, particularly the distinction between somatic and cognitive anxiety symptoms. Given the important role of social support and manifest anxiety in emotional recovery, there is a clear need to investigate factors that predict the development of anxiety in this population. **Objectives:** This study aimed to identify predictors of anxiety and to assess differences between somatic and cognitive anxiety symptoms in children undergoing surgery for lower leg fractures, addressing the need to better understand psychological effects in this vulnerable group. **Methods**: The research included 63 children with lower leg fractures, of whom 40 were boys (63.5%) and 23 were girls (36.5%), with a mean age of M = 15.174 (SD = 3.701). The instruments used in this research were as follows: the Demographic Data Questionnaire, the Children’s Anxiety Scale, the Beck Anxiety Inventory (BAI), and the Multidimensional Scale of Perceived Social Support. **Results**: The results showed that the only significant predictor of somatic symptoms of anxiety was the presence of paresthesia, while significant predictors of cognitive symptoms of anxiety were social support from friends and the presence of paresthesia. **Conclusions**: Paresthesia was identified as a significant predictor of somatic symptoms of anxiety, while social support from friends was associated with lower levels of cognitive anxiety symptoms in children with lower leg and ankle fractures. These results point to the relevance of considering both somatic and psychological factors in the recovery process following pediatric fractures.

## 1. Introduction

Most fractures in childhood, including lower leg fractures, present a significant clinical challenge, especially when complicated and requiring surgical treatment. Lower leg fractures are among the most common fractures in the pediatric population and rank third in frequency by location, after forearm and femur fractures [1]. Hospitalizations of children due to lower leg fractures account for 21.5% of all pediatric hospitalizations [2]. The lower leg may be fractured proximally, in the middle, or distally, affecting the fibula, tibia, or both bones simultaneously. Each fracture, regardless of the level, is specific and requires an individualized therapeutic and rehabilitation approach, making the recovery period uniquely specific for each patient. Proximal lower leg fractures are often associated with longer movement limitations and a tendency to develop contractures, unlike mid-shaft fractures, which mostly do not complicate with knee or ankle contractures, but may have prolonged healing due to a lack of vascularization [3]. Fractures near the ankle may require extended immobilization and result in limited ankle movement, potentially affecting normal gait biomechanics.

When discussing lower leg fractures requiring surgery, regardless of location, each represents an added source of stress for the child. The trauma itself is stressful, further intensified by hospitalization, anesthesia, surgery, postoperative recovery, and ultimately, early and sometimes permanent functional limitations in daily activities.

In addition to physical consequences, children who have suffered fractures—whether treated conservatively or surgically—are at an increased risk of developing psychological and emotional difficulties, as indicated by numerous researchers [4,5,6,7,8]. A frequently present symptom that often receives insufficient attention is the development of anxiety, which can manifest through various somatic symptoms such as rapid breathing, tension, gastrointestinal issues, and insomnia, as well as cognitive symptoms like increased worry, difficulty concentrating, irritability, and a negative anticipation of painful or unpleasant medical procedures. Annually, in the United States alone, 4 million children undergo surgery, and more than half develop fear and anxiety before the procedure itself [9]. The psychological burden can be further exacerbated by feelings of a loss of control, fear of re-injury, isolation from peers and daily activities, as well as the cessation of active training and sports competitions [5,10,11,12,13].

The initial fear is adaptive, but in many children, it can persist after surgery, especially when the child is still partially immobilized for some time after surgery [14]. Stress in this situation can manifest itself in various ways and can involve somatic, cognitive, emotional, and behavioral symptoms [15,16]. Instead, it can also persist for some time and manifest itself as clinical anxiety that extends into the postoperative and rehabilitation phases [17].

For this reason, social support is a key protective factor in preserving children’s mental health during recovery [12,13,18]. Emotional support and the availability and engagement of both parents and close relatives, as well as friends and peers, can significantly contribute to the preservation of children’s mental health by alleviating their fears and feelings of insecurity. Therefore, every rehabilitation plan should include social support to contribute to better emotional regulation in the child and enable a higher-quality recovery.

In this paper, for the purpose of a more precise understanding, we will decompose anxiety into its specific components: cognitive and somatic anxiety, and relate them to the concept of manifest anxiety.

However, data on the interaction of social support with anxiety dimensions (somatic vs. cognitive symptoms) in children undergoing specific medical interventions such as lower extremity surgery are limited. It must be understood that somatic and cognitive symptoms of anxiety in the treatment process are important because these two dimensions may be based on different psychological mechanisms, but the approach itself is different and may have a different relationship with social support. Somatic symptoms of anxiety include a rapid heartbeat, muscle tension, restlessness, and somatic complaints such as dizziness or stomach discomfort [19]. The onset of these types of anxiety symptoms in children may result in feelings of danger or failure, which may in turn lead to increased anxiety [19].

On the other hand, cognitive symptoms of anxiety include worry, negative thoughts, and ruminative behavior characterized by an anxious fear of future events. Cognitive anxiety in children can significantly hinder their functioning across various domains, including their academic performance, social interactions, and overall well-being [20,21]. Recognizing the differences and relationships between the above constructs of anxiety would contribute to the possibility of planning possible psychological interventions that can be different. On the one hand, relaxation and sensorimotor techniques are more useful for expressed somatic symptoms; cognitive symptoms require cognitive–behavioral approaches and parent education [21,22]. Also, as the constructs of anxiety are different, their inter-relationship with social support, which is unexplored, can have a specific effect on certain dimensions of anxiety. Therefore, a better understanding of the relationship between social support and separate dimensions of anxiety can contribute to the development of more targeted psychological assistance programs and facilitate the psychological recovery of children after orthopedic surgery. This is particularly important because after such surgeries, children may feel isolated from society for a certain period of time and from school and important people in their lives because they are prevented from moving, especially over long distances. For this reason, a feeling of rejection may occur [23], which may lead to increased anxiety, which may have already arisen due to the surgery itself.

Manifest anxiety, on the other hand, reflects a person’s general tendency to experience anxiety in different situations and contexts, that is, it is a general, stable tendency of a person to experience increased anxiety in different situations [24]. It can be viewed as a broader, global construct from which specific cognitive and somatic symptoms of anxiety arise [25]. The general level of anxiety can, therefore, be expressed through different channels, either predominantly through mental worry or through somatic reactions [25]. Manifest anxiety can, therefore, be viewed as a predisposing factor that modulates the child’s emotional response to surgery, either through cognitive patterns (e.g., worry, rumination) or somatic symptoms (e.g., tension, physical discomfort). However, it is not yet sufficiently known to what extent manifest anxiety is associated with specific forms of anxiety symptoms in the postoperative period in children, and the results could shed light on these inter-relationships and contribute to a better understanding of recovery in this group.

Therefore, the lack of research on these topics can certainly clarify the inter-relationships of the above constructs that may be important for postoperative psychological recovery in children.

### Objectives

The aim of this study was to identify clinical, sociodemographic, and psychosocial predictors of cognitive, somatic, and manifest anxiety in children after lower extremity surgery, with a special emphasis on the role of social support and the presence of paresthesias in explaining individual dimensions of anxiety.

## 2. Materials and Methods

### 2.1. Population and Sample

During 2024, a cross-sectional study was conducted at the Clinic for Pediatric Surgery, University Hospital Centre Osijek. Data on patients were collected from the hospital information system from 2018 to 2023 regarding the type of fracture and duration of physical therapy in order to create a database of respondents who were invited for check-ups and surveyed during 2024. A total of 63 children aged 4 to 18 years, who underwent various types of surgical treatment for proximal, mid-shaft, or distal lower leg fractures, were included. This study included 40 boys (63.5%) and 23 girls (36.5%), with a mean age of M = 15.174 (SD = 3.701).

### 2.2. Methods

Data were collected in two ways. The first involved reviewing medical records and the hospital information system for fracture type, duration of immobilization, duration of physical therapy, and presence of paresthesia and pain. The second part of the data was collected during the last follow-up examination. Parents were instructed that the Children’s Anxiety Scale should be completed with parental assistance for children under 9 years, while older children completed it independently; the Beck Anxiety Inventory was completed with parental help for children under 16 and independently for older children; and the Multidimensional Scale of Perceived Social Support was completed with parental help for children under 9 and independently for older children. All parents had to provide consent for their children’s participation.

Inclusion criteria encompassed children under 18 years of age who sustained fractures and were treated as acute patients at the Department of Pediatric Surgery, provided that voluntary consent for participation was obtained from both parents and the children themselves.

Exclusion criteria included patients aged 18 years or older, children with pathological lower leg fractures, fractures resulting from genetic diseases, and children with metabolic syndromes. Furthermore, parents and children who did not provide their consent to participate were excluded from this study.

### 2.3. Materials

The questionnaire consisted of five parts: The first part collected data on the child’s sex and age. The second part collected data on fracture type, duration of immobilization, duration of physical therapy, presence of paresthesia, and pain in the leg after the fracture, based on medical records.

Anxiety Scale for Children—used to measure manifest anxiety, which contains 13 items. The scale has three possible answer types: “Yes”, “No”, and “?”. The total score is the sum of all “Yes” answers. The possible range of scores was from 0 to 13 points on the scale, while a higher total score means a higher level of anxiety. The reliability coefficient of the scale in this study was α = 0.67 [26].

Beck Anxiety Inventory (BAI): Consists of 21 items, each rated from 0 to 3, with 0 indicating the symptom does not occur and 3 indicating it is very pronounced. The scale measures cognitive and somatic anxiety symptoms. Higher scores indicate greater anxiety intensity. The total result is calculated as the average of the sum of all answers related to a particular scale and can range from 0 to 3. Reliability coefficient for somatic symptoms: α = 0.89; for cognitive symptoms: α = 0.81 [27,28].

Multidimensional Scale of Perceived Social Support: Measures perceived support from three sources: family, friends, and significant others. The scale consists of 12 items, with four statements for each subscale. Responses are on a 7-point Likert scale from “1” (strongly disagree) to “7” (strongly agree). The possible range of scores for each subscale is from 1 to 28. Reliability coefficients: significant others α = 0.75, family α = 0.82, and friends α = 0.80 [29].

### 2.4. Statistical Methods

Descriptive statistics were used to describe the distribution of variables. The Shapiro–Wilk test was used to test normality of numerical variables (anxiety components, manifest anxiety, and social support components); it was not significant (*p* > 0.05), so parametric tests were used. Means were expressed as arithmetic mean (M), range, and standard deviation (SD). The *t*-test for dependent samples was used to test differences between two dependent variables. Linear regression analysis was used to examine predictors of somatic and cognitive anxiety symptoms. All key conditions for regression were checked and met: linearity, normality of residuals, homoscedasticity, absence of multicollinearity (VIF within acceptable limits), and absence of autocorrelation (Durbin–Watson statistics). Variables that were previously shown to be significant in the bivariate analysis conducted by Spearman were included in the linear regression and Point Biserial correlations were used as appropriate. Analyses were performed using available data. Respondents with missing values in certain variables were not included in certain analyses (listwise deletion) and data imputation was not performed due to the small number of missing values and their random nature. Before conducting statistical analyses, the presence of univariate and multivariate outliers was checked. Individual outliers were identified (1 to 3 per analysis), but after additional evaluation, it was decided to keep them in the analyses because they did not have a significant impact on the results, nor did they deviate to a degree that would statistically justify their exclusion. This preserves the representativeness of the sample and the completeness of the data.

Statistical significance was set at *p* < 0.05. Data were processed using JASP, version 0.19.3.

## 3. Results

The most common type of fracture was the distal tibia and fibula (50.8%). Most children had immobilization for more than five weeks (51.6%) and attended physical therapy for up to two weeks (44.6%). Most did not have paresthesias (76.7%) or leg pain after the fracture (76.7%). The mean value of manifest anxiety was 1.45 (SD = 1.85) and social support from significant others, family, and friends ranged from 24.29 to 26.03. Somatic anxiety symptoms were significantly associated with manifest anxiety (ρ = 0.365, *p* < 0.001), while cognitive symptoms did not show a significant association (ρ = 0.144, *p* = 0.272). Both types of anxiety symptoms were negatively associated with social support from family and friends (*p* < 0.01). The male gender, fracture type, presence of paresthesias, and pain were significantly associated with higher levels of somatic and cognitive anxiety symptoms (Table 1).

Somatic symptoms of anxiety were statistically significantly higher than cognitive symptoms in children after surgery for lower leg and ankle fractures (*p* = 0.013; Cohen’s d = 0.32), indicating a small-to-moderate effect size difference (Table 2).

In order to determine the predictors of somatic symptoms of anxiety in children undergoing different types of surgery for fractures of the lower leg and ankle, a linear regression analysis was conducted. The analysis included variables that were found to be significant in correlations (the type of fracture, dimensions of social support from family and friends, gender, presence of paresthesia, and pain). The results showed that the group of variables included in the analysis significantly explained 43.9% of the variance of somatic symptoms of anxiety, which represents a medium-to-strong effect size (adjusted R^2^ = 0.439; *p* < 0.001). Only the variable of the presence of paresthesia was shown to be a significant predictor (*p* = 0.013) and an insight into the β coefficient shows that the presence of paresthesia contributes to greater somatic symptoms of anxiety (Table 3).

In order to determine the predictors of cognitive symptoms of anxiety in children undergoing different types of surgery for fractures of the lower leg and ankle, a linear regression analysis was conducted. The analysis included variables that were found to be significant in the correlations (dimensions of social support from family and friends, gender, the presence of paresthesia, and pain). The results showed that the group of variables included in the analysis significantly explains 40.2% of the variance of the somatic symptoms of anxiety, which represents a medium-to-strong effect size (adjusted R^2^ = 0.402; *p* < 0.001). The variables of the presence of paresthesia (*p* = 0.049) and the dimension of social support from friends (*p* = 0.025) were shown to be significant predictors and an insight into the β coefficient shows that the presence of paresthesia and lower social support from friends contribute to greater cognitive symptoms of anxiety (Table 4).

## 4. Discussion

The presented research was conducted as part of a cross-sectional study conducted at the Clinic of Surgery, Department of Pediatric Surgery, KBC Osijek. In this study in which 63 children participated, the majority (63.5%) were boys, as in most studies conducted on the population of children with lower leg fractures [30,31,32,33]. The most common type of fracture in this study was a fracture of the distal part of the tibia and fibula, 50.8%, which is partly in line with a study conducted in a hospital in Switzerland with the aim of investigating the epidemiology and injury pattern of lower limb fractures in children, which showed that the most common fractures are in the distal third of the lower leg, as many as 74% [34]. The obtained result could be explained in several ways. Namely, according to the anatomical structure, the distal part of the lower leg is thinner and more sensitive than the proximal and diaphyseal parts and is also less protected by the musculature; therefore, it is less resistant to impacts and torsional forces. In torsion injuries, which are particularly common in sports activities, the ankle joint, which is very mobile, twists, and these forces are transferred to the more fixed parts of the distal tibia and fibula, resulting in bone injuries in that part. This study also showed that children with distal fractures (closer to the ankle) have more pronounced symptoms of anxiety, while those with proximal fractures (closer to the knee) have less pronounced symptoms. The above result could be interpreted as children probably experiencing distal fractures as more stressful, either due to more pronounced pain or a greater impact on mobility. Distal fractures are mostly more complex, require a more complex procedure, and often the limitation of movement persists for a longer time compared to proximal fractures. All of the above leads to prolonged limitations in both activities of daily living and social activities such as playing with peers, which likely leads to more pronounced physical reactions of anxiety.

The length of immobilization in most children included in the study was 5 weeks or more, which is a result of the complexity of the fracture and monitoring the healing rate through control radiological images. The duration of physical therapy in most subjects (44.6%) was up to 2 weeks (including the second week), while in 28.6%, physical therapy lasted 3–4 weeks. It should be taken into account that the length of physical therapy and rehabilitation also depends on the time when it was started because in subjects included in this study, it was often not possible to start prompt physical therapy after removing the immobilization. This study showed that somatic symptoms of anxiety were significantly greater than cognitive symptoms and that somatic symptoms of anxiety are significantly associated with manifest anxiety. The above results could be explained in a way that somatic symptoms are much more easily recognizable in children than cognitive ones because children are often not able to verbalize their concerns as adults can, do not have a fully developed way of thinking about abstract threats, and do not focus on future consequences. Traumatic experiences such as bone fractures and surgery can provoke manifest anxiety in response to stressful events, and children can often react with visible symptoms of stress such as rapid breathing, trembling, crying, nervousness, or withdrawal. Previous studies have shown that more than 50% of children undergoing surgery in the United States develop significant behavioral stress and anxiety even before surgery [35], and their significant impact on both postoperative anxiety and overall recovery has been shown [36,37]. Research by Forthier and colleagues has also shown that the individual characteristics of each child significantly contribute to the development of postoperative behavior, so that awareness of individuality by parents and physicians is very important in order to prevent or reduce difficulties such as anxiety and depression in the postoperative period [38].

### 4.1. Social Support and Anxiety

The results of this study have shown that social support has a positive effect on preventing and reducing anxiety symptoms. Children who feel more support from family and friends have less pronounced symptoms of both somatic and cognitive anxiety.

Previous studies have shown that children and their parents develop anxiety symptoms in the preoperative period, which predispose them to the development of a number of negative postoperative outcomes, such as slower postoperative recovery, a greater need for analgesics [39,40], and a threefold higher risk of developing postoperative anxiety [41]. Considering the possible negative consequences of anxiety, clinicians and researchers have tried to apply various interventions such as preoperative sedation, psychological programs, and therapies such as reading picture books or age-appropriate content, all with the aim of reducing the frequency, intensity, and consequences of anxiety [42,43,44]. Recent Cochrane reviews of non-pharmacological interventions for children’s preoperative anxiety conclude that non-pharmacological interventions such as the clown doctor method, hypnosis, and handheld video games are as effective as pharmacological interventions in reducing anxiety and improving children’s cooperation [45,46]. It has also been found that modern audiovisual interventions, such as the use of computers, video glasses, and various mobile phone applications, can be useful in preparing children and parents for surgical procedures and reduce preoperative anxiety and its later consequences [47,48]. A systematic review of studies supporting the use of audiovisual interventions for reducing anxiety in children in the preoperative period concludes that these interventions have been shown to be even more effective than standard care, which is still insufficiently available, in reducing anxiety [49]. Therefore, audiovisual interventions could become an attractive and cost-effective solution, with indispensable social support, in reducing both preoperative anxiety and its consequences. Namely, children who have the support of close people in the form of parents and friends simply being present or providing support through conversation develop their emotional security and alleviate or prevent the development of cognitive symptoms of anxiety such as tension, fear, or worry. The presence of friends and family as well as a sense of belonging to a group help children focus on positive things and thoughts through play and conversation, thus distancing themselves from the role of the patient, returning more easily to daily activities and routines, and reducing internal tension and anxious thoughts. Children who do not have adequate social support from peers, especially if they are excluded from social activities and sports that were important to them before surgery, often develop feelings of isolation, insecurity, and reduced value. The results of this study are in line with previous research that confirmed that greater social support acts as a protective factor, provides a sense of security and emotional stability, and alleviates the somatic and mental consequences of stress, especially in sensitive situations such as recovery from surgery [50,51,52,53]. These findings indicate the need for the further development and improvement of protocols in everyday practice that would encourage social support programs in hospitals and rehabilitation centers, including parents, friends, and peers, in order to achieve the fastest possible emotional and functional recovery and return to activities of daily life. It is of the utmost importance to educate parents to recognize different emotional states in a child, accept them, and provide the child with timely and adequate positive encouragement in establishing and maintaining emotional stability. It is also necessary to emphasize the role of the school system as a very important factor in organizing the integration of a child after recovery. In conclusion, the inclusion of the entire social environment, whether in person or through digital channels, in all phases of a child’s recovery is a key step in order to minimize psychological consequences, maintain mental health, and encourage and achieve successful rehabilitation.

### 4.2. Paresthesia and Anxiety

The results of this study showed that the presence of paresthesia and pain significantly positively correlates with both somatic and cognitive symptoms of anxiety, and the presence of paresthesia has been shown to be a significant predictor of the development of greater anxiety symptoms. Paresthesias are abnormal sensory experiences and are usually described as tingling, burning, numbness, or a feeling of swelling and arise due to changes in the nervous system that may be associated with damage to autonomic nerve fibers or changes in the transmission of nerve impulses [54]. They can be confusing and frightening in children who cannot verbalize them and who are not fully aware of these causes, as well as increasing the stress response in the body because the nervous system can become hypersensitive to stimuli. The child’s existing psychological vulnerability, caused by trauma, can be further increased by the stress caused by paresthesia, which makes anxiety symptoms more intense. Paresthesias can occur as a result of the trauma itself where the nerve was compressed or after surgery on the lower leg as a result of irritation or damage to the nerve during the surgery itself. Postoperative swelling or immobilization can also be the cause of the greater or lesser compression of the nerve, causing unpleasant sensations. Children can also associate the presence of paresthesias with the traumatic and painful experience of surgery, which can have a retraumatizing effect and increase anxiety, feelings of insecurity, and withdrawal, and it is, therefore, very important to alleviate them as early and adequately as possible.

Paresthesias were identified in the results of this study as a strong predictor of somatic and cognitive symptoms of anxiety, which indicates the need for the timely and regular assessment of not only the motor and functional, but also sensory and neurological status during the rehabilitation process. In future clinical practice and rehabilitation protocols, the evaluation of sensory symptoms should certainly be expanded and improved because their timely recognition, alleviation, or elimination can accelerate functional recovery and prevent the development of serious emotional and psychological disorders. A very important measure in these protocols should be the education of parents about possible neurological disorders and their impact on the psychological health of children, with the aim of the early recognition of verbal and nonverbal signs that children show in response to paresthesias and other neurological symptoms.

The results of this study ultimately emphasize the importance of the timely recognition and treatment of somatic symptoms of anxiety in children after fracture surgery, especially in those with distal fractures and present paresthesias. In clinical practice, it is important to pay attention to somatic signs of anxiety, which children more easily express than cognitive symptoms. Also, early intervention through appropriate pain control, early physical therapy, and emotional support provided from family and friends can significantly reduce the development of anxiety and facilitate recovery. These findings indicate the need for an individualized approach to each child in postoperative treatment, with an emphasis on the overall physical and psychological condition.

## 5. Conclusions

It is important to recognize that lower leg fractures, although seemingly an orthopedic problem, also pose a significant challenge to the child’s mental health. In conclusion, this study conducted on children with lower leg and ankle fractures strongly emphasizes the importance of paresthesia as a significant predictor of both somatic and cognitive symptoms of anxiety. Furthermore, the findings highlight the importance of social support from friends as a predictor of lower levels of cognitive anxiety. These results clearly indicate that recognizing paresthesia as a potential risk factor and encouraging and ensuring social support from friends can reduce anxiety and improve the psychological well-being of children during fracture recovery. However, further research is needed to understand the psychological aspects and inter-relationships of the above constructs and symptoms in order to provide children with comprehensive and tailored care, with the goal of complete physical and emotional rehabilitation.

## 6. Study Limitations

The conducted research, like many others, has its shortcomings, which is why the data obtained must be interpreted with caution. Namely, despite the age-appropriateness of the questionnaire for the respondents, there is a possibility that younger respondents (despite the explanation by the parents) did not fully understand the meaning of all parts or were not aware of their own reactions, behavior, or emotions. It is also possible that parents did not fully recognize certain manifest behaviors in younger children that could indicate difficulties, or even that they minimized their occurrence or attributed them to some other factor. Considering that self-assessment measures were used, it is possible that some participants, due to the sensitivity of certain questions, gave socially desirable answers in which they tried to present themselves in a better light. An important limiting factor is also the aspect of the participants’ recollection of their own experience and functioning when filling out the questionnaire, especially in younger children who have a poorer memory and are more susceptible to suggestion.

It is also important to note that the research was conducted on a relatively small number of respondents and within a single health institution. Such limitations may affect the possibility of generalizing the obtained results to a wider population. A sample that is not large enough and geographically diverse may have specific characteristics that are not representative of children with lower leg and ankle fractures in the general population. Therefore, the results should be interpreted with caution, with the recommendation that future research include a larger number of participants from different backgrounds to increase the external validity of the findings.

## Figures and Tables

**Table 1:** Sample characteristics, descriptive statistics of anxiety and social support variables, and their associations with demographic and clinical factors.

				*n* (%)
Type of fracture	diaphysis of tibia and fibula			18 (28.6)
	distal part of tibia and fibula			32 (50.8)
	proximal part of tibia			8 (12.7)
	bilateral fractures of distal metaphysis of lower leg			5 (7.9)
Length of immobilization *n* = 62	up to 3 weeks			7 (11.3)
	4 weeks			23 (37.1)
	5 and more weeks			32 (51.6)
Duration of physical therapy *n* = 56	up to 2 weeks			25 (44.6)
	3–4 weeks			16 (28.6)
	5–6 weeks			6 (10.7)
	more than 6 weeks			9 (16.1)
Paresthesia *n* = 43	yes			10 (23.3)
	no			33 (76.7)
Pain in the leg after fracture *n* = 43	yes			10 (23.3)
	no			33 (76.7)
	**M ± SD**			
Manifest Anxiety	1.450 ± 1.854			
Social Support—Significant Others	25.661 ± 3.318			
Social Support—Family	26.031 ± 3.232			
Social Support—Friends	24.285 ± 3.756			
		**Somatic Symptoms of Anxiety**	**Cognitive Symptoms of Anxiety**	
Manifest anxiety	ρ	0.365	0.144	
	*p*	**<0.001**	0.272	
Social Support—Significant Others	ρ	−0.198	−0.156	
	*p*	0.122	0.226	
Social Support—Family	ρ	−0.471	−0.385	
	*p*	**<0.001**	**0.002**	
Social Support—Friends	ρ	−0.387	−0.362	
	*p*	**0.002**	**0.004**	
Age	ρ	−0.084	−0.021	
	*p*	0.513	0.868	
Gender	r_PB_	0.381	0.392	
	*p*	**0.002**	**0.002**	
Type of fracture—diaphysis of tibia and fibula	r_PB_	−0.031	0.012	
	*p*	0.808	0.928	
Type of fracture—distal part of tibia and fibula	r_PB_	0.278	0.152	
	*p*	**0.027**	0.235	
Type of fracture—bilateral	r_PB_	−0.132	−0.004	
	*p*	0.303	0.975	
Type of fracture—proximal part of tibia	r_PB_	−0.268	−0.241	
	*p*	**0.033**	0.057	
Presence of paresthesia	r_PB_	0.481	0.374	
	*p*	**0.001**	**0.013**	
Pain	r_PB_	0.460	0.458	
	*p*	**0.002**	**0.002**	
Length of immobilization	ρ	−0.138	−0.118	
	*p*	0.287	0.360	
Duration of physical therapy/rehabilitation	ρ	0.217	0.103	
	*p*	0.108	0.448	

Note: *n*—number of respondents; %—percentage; M—Mean; SD—Standard Deviation; *p*—Statistical significance; r_PB_—Point-biserial correlation coefficient; ρ—Spearman correlation coefficient.

**Table 2:** Descriptive statistics and differences between somatic and cognitive anxiety symptoms.

	M ± SD	Cohen’s d	*p* *
Somatic Symptoms of Anxiety	0.415 ± 0.445	0.324	**0.013**
Cognitive Symptoms of Anxiety	0.338 ± 0.385		

Note: SD—Standard deviation; *p*—Statistical significance; * *t*-test.

**Table 3:** Results of regression analysis—somatic symptoms of anxiety-dependent variable.

	β	t	*p*	Adjusted R^2^
(Constant)		1.316	0.198	0.439
Manifest anxiety	0.006	0.038	0.970	
Social Support—Family	−0.039	−0.248	0.806	
Social Support—Friends	−0.204	−1.189	0.244	
Gender	0.188	1.367	0.181	
Type of fracture—distal part of tibia and fibula	0.115	0.713	0.481	
Type of fracture—bilateral	0.040	0.326	0.747	
Type of fracture—proximal part of tibia	−0.191	−1.372	0.180	
Presence of paresthesia	0.358	2.645	**0.013**	
Pain	0.221	1.438	0.160	

Note: β—regression coefficient; t—the magnitude of the difference in relation to the variation in the sample data; *p*—statistical significance; adjusted R^2^—coefficient of determination.

**Table 4:** Results of regression analysis—cognitive symptoms of anxiety-dependent variable.

	β	t	*p*	Adjusted R^2^
(Constant)		3.355	0.072	0.402
Social Support—Family	0.027	0.195	0.846	
Social Support—Friends	−0.380	−2.331	**0.025**	
Gender	0.226	1.758	0.087	
Presence of paresthesia	0.252	2.037	**0.049**	
Pain	0.171	1.194	0.240	

Note: β—regression coefficient; t—the magnitude of the difference in relation to the variation in the sample data; *p*—statistical significance; adjusted R^2^—coefficient of determination.

## Data Availability

The data are available from the corresponding author upon reasonable request.
